# Comparison of Motion Artefact Reduction Methods and the Implementation of Adaptive Motion Artefact Reduction in Wearable Electrocardiogram Monitoring

**DOI:** 10.3390/s20051468

**Published:** 2020-03-07

**Authors:** Xiang An, George K. Stylios

**Affiliations:** Research Institute for Flexible Materials, Heriot-Watt University, Edinburgh, Scotland EH14 4AS, UK; xa30@hw.ac.uk

**Keywords:** electrocardiogram, motion artefact, adaptive filtering, impedance pneumography signal

## Abstract

A motion artefact is a kind of noise that exists widely in wearable electrocardiogram (ECG) monitoring. Reducing motion artefact is challenging in ECG signal preprocessing because the spectrum of motion artefact usually overlaps with the very important spectral components of the ECG signal. In this paper, the performance of the finite impulse response (FIR) filter, infinite impulse response (IIR) filter, moving average filter, moving median filter, wavelet transform, empirical mode decomposition, and adaptive filter in motion artefact reduction is studied and compared. The results of this study demonstrate that the adaptive filter performs better than other denoising methods, especially in dealing with the abnormal ECG signal which is measured from a patient with heart disease. In the implementation of adaptive motion artefact reduction, the results show that the use of the impedance pneumography signal as the reference input signal for the adaptive filter can effectively reduce the motion artefact in the ECG signal.

## 1. Introduction

The electrocardiogram (ECG) is a weak continuous signal with the amplitude of its potential ranging from 0.5 mV to 5 mV [1,2]. ECGs are often recorded with different types of noise such as power line interference, muscle noise, and motion artefact. Among all these noise types, the motion artefact is the most difficult type of noise to eliminate because its spectrum usually overlaps with the very important spectral components of the ECG signal, making it difficult for traditional signal processing techniques to separate. Therefore, the motion artefact has long been a problem in ECG measurement, especially for exercise ECG and ambulatory ECG recording.

Motion artefacts are transient (but not step) baseline wander resulting from movement of the patient or the electrode [3]. Tam and Webster [4] have demonstrated that physical abrasion of the skin under the ECG electrodes with sandpaper is an effective way to reduce motion artefacts. However, for long-term ambulatory ECG measurements, skin abrasion is not recommended because the skin regenerates over time, resulting in increased skin potential. Moreover, skin abrasion in fact causes damage to skin, making it unsuitable for people with sensitive or fragile skin conditions.

Over the last few decades, traditional signal-processing techniques such as finite impulse response/infinite impulse response (FIR/IIR) high-pass filtering, moving average filtering, and wavelet transform have been used to reduce the motion artefact [5,6,7,8,9]. Among all these noise-reduction methods, an IIR high-pass filter with a cut-off frequency of 0.5/0.67 Hz is one of the most common methods used in practice today [10,11]. But this method has a drawback that the 0.5 Hz or 0.67 Hz low-frequency cut-off filter can introduce considerable distortion in the ECG signal, particularly with respect to the T wave and the level of the ST segment [12,13,14,15]. Other methods such as the moving average filter [16,17] and the moving median filter [18,19] have also been used in motion artefact reduction, but the performance of these methods is sensitive to the selection of window length. Some researchers have also used discrete wavelet transforms to suppress noise [20,21,22,23,24,25], but the selection of appropriate wavelet function and threshold plays an important role in signal denoising. Empirical mode decomposition (EMD) was also proposed for baseline wander reduction [26,27,28,29], but this method requires a priori knowledge of baseline wander behavior. In recent years, adaptive filtering has been proven to be useful in motion artefact reduction [30].

In this article, we have studied different signal-processing techniques and compared their performance for motion artefact reduction, to enable ECG monitoring during moving and exercising, and we have implemented the best-performing technique during ECG monitoring. Because the adaptive filter has the ability to adaptively track the signal under body-moving conditions, as in the case of ECG, it is theoretically more suitable for reducing motion artefacts in the ECG signal than other signal-processing methods. In order to discuss the possibility of using adaptive filter for motion artefact reduction in practice, we designed hardware that supports simultaneous recording of ECG and impedance pneumography (IP) signals. A preliminary experiment is undertaken with the designed hardware to demonstrate that the adaptive filter can successfully reduce motion artefact in practice when the reference input signal has a good correlation with the motion artefact to be cancelled.

## 2. Motion Artefact Reduction Methods

### 2.1. High-Pass Finite Impulse Response (FIR) Filter

Digital filters are broadly divided into two classes: finite impulse response (FIR) and infinite impulse response (IIR). For ECG interpretation and diagnosis, the measured ECG needs to have a good low-frequency response to reproduce the ST segments accurately. In order to reduce artefactual distortion of the ST segment, the 1990 American Heart Association (AHA) document recommended that “the low-frequency cut-off be 0.05 Hz for routine filters but that this requirement could be relaxed to 0.67 Hz or below for linear digital filters with zero phase distortion” [31]. In this recommendation, the requirement of linear digital filters with zero phase distortion is critical because if digital filters have non-linear phase response, phase distortion is introduced into the signal, resulting in distortion of the low-frequency components of the ECG signal. The primary advantages of FIR filters are that they have linear phase response and are always stable, while the IIR filters have non-linear phase response, so the FIR filters are commonly used for biomedical signal processing.

FIR filtering can be described by the following discrete difference equation:

(1) $$y\left(n\right)=\overset{M-1}{\underset{m=0}{\sum }}b_{m}x\left(n-m\right)$$

where $b_{m}$ is the coefficient of the filter, *M* − 1 is the order of the filter, *M* is the length of the filter, x(n) is the input signal, y(n) is the filtered signal. The FIR coefficient $b_{m}$ can be calculated by the Window method [32], and the selection of the window function should first satisfy the passband and attenuation requirements, and then determine the number of filter coefficients based on the relationship between the filter length and the transition width.

**Figure 1:**
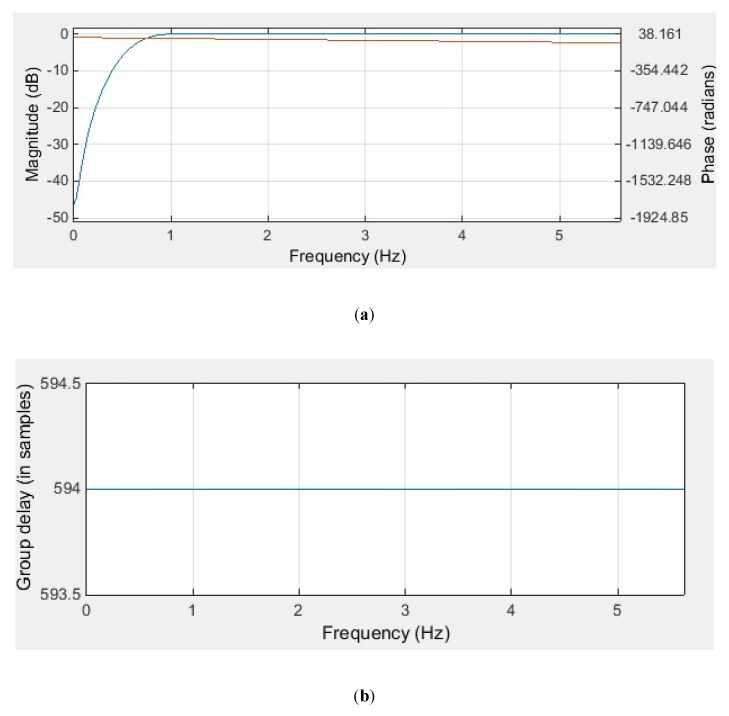
The designed finite impulse response (FIR) high-pass filter: (**a**) magnitude response (blue) and phase response (red); (**b**) group delay.

In our study, we used the Hamming window function to design a high-pass FIR filter. The Hamming window function has a relatively small transition width and a higher stopband attenuation than other window functions. In order to suppress low-frequency motion artefacts, the cut-off frequency ($f_{c}$) of the high-pass FIR filter is selected to be 0.5 Hz. Since the transition width of the Hamming window is $3.3f_{s}/M$, the required filter order (*M* − 1) is at least 3.3$f_{s}$. When the sampling frequency ($f_{s}$) of the ECG signal is 360 Hz, the required filter order should be at least 1188. The filter coefficients were calculated using the Filter Design and Analysis (FDA) tool of MATLAB software, and the characteristics of the designed FIR filter are shown in Figure 1.

As demonstrated in Figure 1, the designed FIR filter has a transition width of 1, which means that not only the filter will suppress signal components in the frequency range of 0–0.5 Hz, but the amplitude of signal components between 0.5–1 Hz will also be affected by the filter. Increase filter order can help to reduce the transition width, but the increased filter order also causes greater time delays in signal processing. In our experiment, because the filter order of the designed FIR filter is 1188, this filter will cause a delay of 1.65 s. If we increase the filter order to reduce the transition width, the time delay caused by the filter will be greater than 1.65 s. This is the disadvantage of FIR filters when filtering low-frequency noise, such as motion artefacts of ECG signals. However, because the amplitude suppression is not obvious in the frequency range of 0.5–1 Hz, the filtered ECG signal does not affect the diagnosis of heart rate variability (HRV), but it is not conducive to the diagnosis of myocardial infarction or ischemia that needs to be reflected by the ST segment of the ECG waveform.

### 2.2. High-Pass Infinite Impulse Response (IIR) Filter with Zero-Filtering Technique

IIR filtering can be described by the following discrete difference equation:

(2) $$y\left(n\right)=\overset{M}{\underset{m=0}{\sum }}b_{m}x\left(n-m\right)-\overset{N}{\underset{m=1}{\sum }}a_{m}y\left(n-m\right)$$

where $b_{m}$, $a_{m}$ indicates the coefficients of the filter, *N* is the order of the filter, x(n) is the input signal, and y(n) is the filtered signal. In comparison with FIR filters, IIR filters can use a much lower filter order to meet the required stopband attenuation. However, IIR filters have non-linear phase responses, which means that the time delay of the different frequency components of the input ECG signal will be different after filtering, resulting in distortion of the ECG waveform. In order to overcome this problem, the zero-phase filtering technique should be used with the IIR filter. The zero-phase filtering technique provides zero phase distortion by processing the input signal in both the forward and backward directions: in the forward step the input signal is filtered with the designed filter, and in the backward step the obtained result is flipped in time prior to the filtering with the same filter [33].

The design of the IIR filter can be based on four classical filters: Butterworth filter, Chebyshev Type I filter, Chebyshev Type II filter, and Elliptic filter. The Butterworth filter has monotonic magnitude response in both passband and stopband, the Chebyshev Type I filter has ripple in the passband, the Chebyshev Type II filter has ripple in the stopband, and the Elliptic filter has ripple in both the passband and the stopband. For ECG signal preprocessing, since the desired filter should have a ripple free passband, the Butterworth filter is the best choice among these four types of IIR filter.

**Figure 2:**
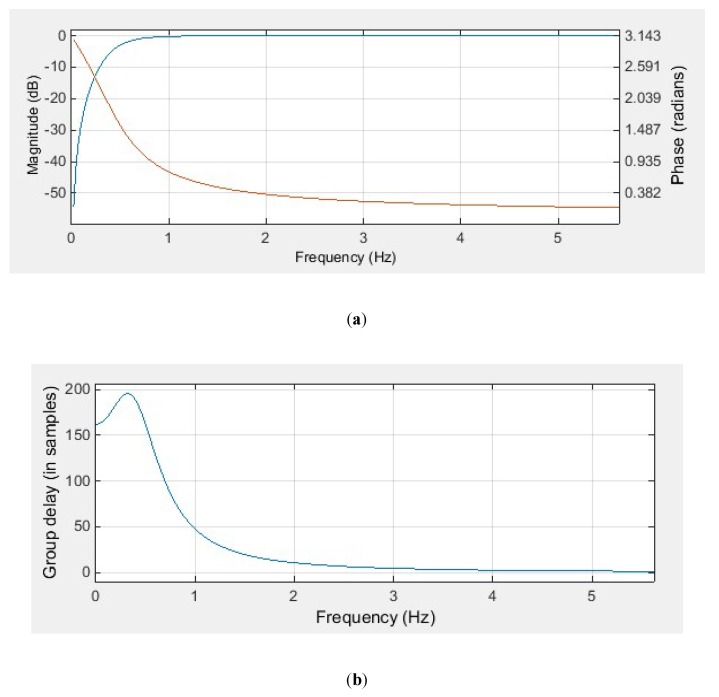
The designed infinite impulse response (IIR) high-pass filter: (**a**) magnitude response (blue) and phase response (red); (**b**) group delay.

In our study, a Butterworth high-pass filter with a cut-off frequency of 0.5 Hz and a filter order of 2 was designed using the FDA tool from the MATLAB software. Figure 2 shows the characteristics of the designed IIR filter.

**Figure 3:**
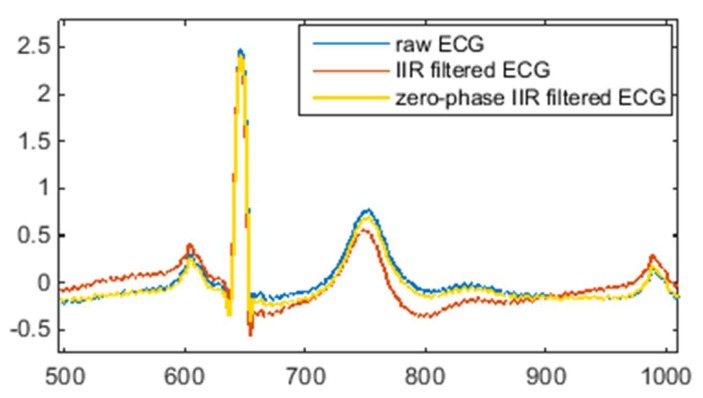
Comparison of conventional and zero-phase infinite impulse response (IIR) filtering.

As demonstrated in Figure 2, the Butterworth filter has a non-linear phase response. Comparing Figure 1b with Figure 2b, the delay caused by the FIR filter and IIR filter is different. For the designed FIR filter, all frequency components of the input signal are delayed in time by the same amount. This kind of delay does not distort the signal waveform, it can be compensated simply by shifting the filtered signal forward by 594 samples. However, for the designed IIR filter, the different frequency components of the input signal have different time delays, especially in the frequency range of 0–1 Hz, which means that the low-frequency components of the ECG signal are distorted after IIR filtering. In order to compensate for the delay and distortion caused by IIR filtering, the zero-phase filtering technique has to be used along with the IIR filter. Figure 3 illustrates the effect of IIR filtering and zero-phase IIR filtering on the shape of the ECG waveform.

As can be seen from Figure 3, IIR filtering causes a certain degree of deformation of the ECG waveform, while zero-phase IIR filtering keeps the morphology of the ECG waveform unchanged. Therefore, in order to suppress motion artefacts and maintain the shape of the ECG signal, the zero-phase technique is critical for high-pass IIR filtering. One major drawback of zero-phase IIR filtering is that it is not suitable for real-time ECG signal processing without a time lag because the zero-phase technique is accomplished with a bidirectional filter by a second filtering pass that is applied in reverse time.

### 2.3. Moving Average Filter

The moving average filter smooths data by replacing each data with the average of its neighboring data. It can be considered as a high-pass FIR filter that has the following form:

(3) $$y_{n}=\frac{1}{M}\overset{\frac{1}{2}\left(M-1\right)}{\underset{i=-\frac{1}{2}\left(M-1\right)}{\sum }}x_{n-i}$$

(4) $$z_{n}=x_{n}-y_{n}$$

where $x_{n}$ denotes a noisy ECG signal, $y_{n}$ denotes the estimated noise, $z_{n}$ is the ECG signal after denoising, *M* is the length of the filter. Moving average filtering is a simple method for low-frequency noise reduction, which has been used in some studies [5,17,34]. However, since the QRS complex of the ECG signal has an extreme amplitude that affects the average, this method may introduce signal distortion.

The moving average filter can be seen as a high-pass filter with a cut-off frequency of fc=fs2M [35], the choice of filter length *M* affects the cut-off frequency of the filter. In our study, because the ECG signal was sampled at 360 Hz, we expected to be able to filter out low-frequency motion artefacts below 0.5 Hz, so the length of the high-pass moving average filter was selected as 361.

### 2.4. Moving Median Filter

The moving median filter is based on the same principle as the moving average filter, in which the median within a moving window of a given length is calculated instead of the average. The moving median filter has also been used to reduce low-frequency noise in some studies [8,36]. In our study, the window length of the moving median filter was chosen to be 361, the same length as the moving average filter, for comparison purposes.

### 2.5. Wavelet Transform Denoising

**Figure 4:**
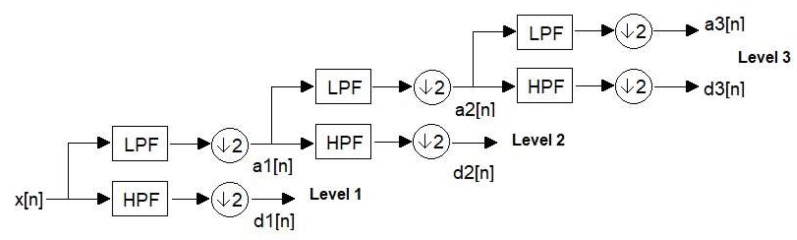
A demonstration of 3-level discrete wavelet transform (DWT) decomposition.

Wavelet-based low-frequency noise cancellation uses the discrete wavelet transform (DWT) to decompose the ECG signal into several time-domain signals at different frequency bands, then sets the approximation coefficients at the lowest frequency band to zero and reconstructs the ECG signal by synthesizing the modified coefficients. As demonstrated in Figure 4, the DWT decomposes the signal *x*(*n*) into detail coefficients *d*(*n*) and approximation coefficients *a*(*n*). The approximation coefficient of the decomposed signal is related to the low-frequency part of the signal, and the detail coefficient of the decomposed signal is related to the high-frequency part of the signal.

In DWT, the choice of wavelet is important for signal denoising performance. There are several commonly used wavelets such as Haar wavelet, Daubechies wavelet and Symlet wavelet. Comparative studies of different wavelets for ECG denoising have been conducted by different researchers. Singh and Tiwari [22] found that the Daubechies wavelet of order 8 (db8) is the most appropriate wavelet basis function for ECG denoising uses. Selijuq et al. [37] suggested that the Daubechies wavelet of order 9 is the most appropriate. Chaudhary et al. [38] found that the Symlet wavelet of order 10 is the optimum combination for ECG denoising. The conclusions of these studies are different mainly because the characteristics of the noise used in these studies are different.

In order to process the noisy ECG with the most appropriate wavelet basis function, we had done some preliminary studies and found that the db8 wavelet is the most suitable wavelet basis function for noisy ECG signals used in our study. To eliminate motion artefacts below 0.5 Hz, we used the db8 wavelet to decompose the noisy ECG signal into 9 levels. The reason for choosing the 9-level decomposition is that when the signal sampling frequency is 360 Hz, the frequency range of the ninth approximation coefficient is about 0–0.7 Hz, which is the frequency range of the noise we want to eliminate. After decomposition, we set the 9th approximation coefficient to zero and reconstructed the ECG signal with all the detail coefficients to obtain the denoised ECG signal.

### 2.6. Empirical Mode Decomposition (EMD)

EMD was introduced by Huang et al. [39] as an analysis method for non-linear and non-stationary signals. It decomposes a signal into a number of intrinsic mode functions (IMF) and a residual signal using the shifting process. An IMF is defined as a function with the same number of extrema and zero crossings, whose envelopes are symmetric with respect to zero. For *N* intrinsic mode functions, the original signal *X*(*n*) can be expressed as:

(5) $$X\left(n\right)=\overset{N}{\underset{i=1}{\sum }}c_{i}\left(n\right)+r_{N}\left(n\right)$$

where $c_{i}\left(n\right)$ represents an intrinsic mode function, and $r_{N}\left(n\right)$ represents the residual signal.

Since EMD decomposes a signal into IMFs, some of which contain only useful signal information, while others contain signal and noise, it can be used for signal denoising in ECG signal processing [26,27,28,29,40]. The levels of decomposition is related to the features of the noise to be suppressed.

In our study, we apply the EMD method to decompose the noisy ECG signal into 7 components which includes 6 IMFs and 1 residue. As the low-frequency noise is only related to the last few components of IMFs, we use the sum of the last 2 IMFs and the residue as the estimation of the noise and then subtract the estimate noise from the noisy ECG signal to obtain the denoised ECG signal.

### 2.7. Adaptive Filter

**Figure 5:**
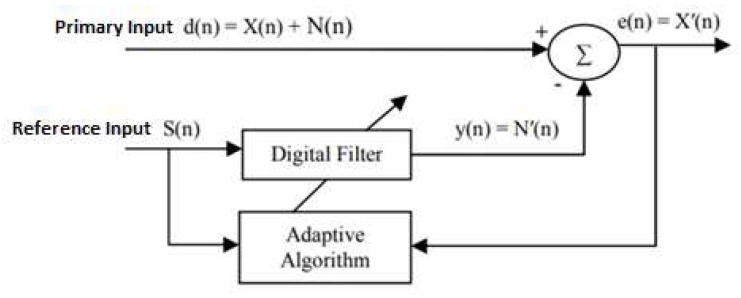
Principle of the adaptive filter in noise cancellation.

An adaptive filter [30] is a time-variant filter which is self-designed and has the ability to adjust its parameters automatically according to an optimization algorithm. It has the capability of adaptively tracking the signal under non-stationary conditions. The adaptive filter can be used for different purposes, such as system identification, prediction, and noise cancellation. The concept of adaptive filtering in noise cancellation is shown in Figure 5.

The adaptive filter requires two sets of input signals: the primary input and the reference input. The primary input signal *d*(*n*) contains both the desired signal *X*(*n*) and noise *N*(*n*). The reference input signal *S*(*n*) is correlated with the noise *N*(*n*) but uncorrelated with the signal *X*(*n*). The reference signal *S*(*n*) is fed into a digital filter to produce an output *y*(*n*), which is as close as possible to the replica of the noise *N*(*n*). The coefficients of the digital filter are continuously changed according to the chosen adaptive algorithm. Subsequently, this filtered signal output *y*(*n*) is subtracted from the primary input *d*(*n*) to obtain the estimated desired signal *X*’(*n*), as demonstrated in the equation:

*e*(*n*) = *X*′(*n*) = *X*(*n*) + *N*(*n*) − *N*′(*n*) (6)

Subtracting noise from the primary input signal involves the risk of distorting the desired signal, and if done improperly it can result in noise level increase. However, if filtering and subtraction are controlled by an appropriate adaptive process, noise reduction can be accomplished with little risk of distorting the signal or increasing the output noise level [30]. An ideal situation is that the noise estimate *N*′(*n*) is an exact replica of the noise *N*(*n*). Since the estimation of *N*′(*n*) is governed by the reference input signal and the adaptive algorithm, the selection of reference input signal and adaptive algorithm are important to the performance of the adaptive noise cancellation. A suitable reference input signal should correlate in some way with the noise that we want to remove but uncorrelated with the “desired” signal [30]. In other words, we need a reference input signal that is highly correlated with the noise in the primary input.

The adaptive filter can be implemented by different adaptive algorithms, but Least Mean Square (LMS), Normalized Least Mean Square (NLMS), and Recursive Least Square (RLS) are the three most commonly used. Every algorithm has its pros and cons. In this study, we use the RLS adaptive filter to process the synthesized noisy ECG because the RLS algorithm provides a faster convergence speed than the LMS and NLMS algorithms.

## 3. Experimental Signals and Evaluation Parameters

To evaluate and compare the noise reduction performance of different signal-processing techniques, most studies use synthetic noisy ECG signals rather than real noisy ECG signals because the calculation of quantitative evaluation parameters requires a “clean” ECG signal. In this study, we have also used synthetic noisy ECG signals to investigate the performance of different denoising methods. The experimental signals were synthesized by adding baseline wander noise to the “clean” ECG signals. The baseline wander noise was used to simulate motion artefacts in the ECG signal. The “clean” ECG signal and the baseline wander noise were obtained from the MIT-BIH database [41,42].

### 3.1. Experimental Signals

**Figure 6:**
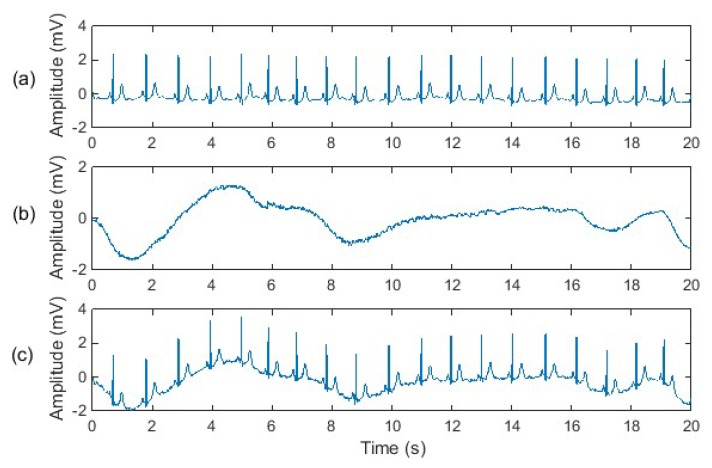
Experimental signals: (**a**) normal electrocardiogram (ECG) from the MIT-BIH database “mitdb/106”; (**b**) baseline wander noise from the MIT-BIH database “nstdb/bw”; (**c**) noisy ECG signal.

**Figure 7:**
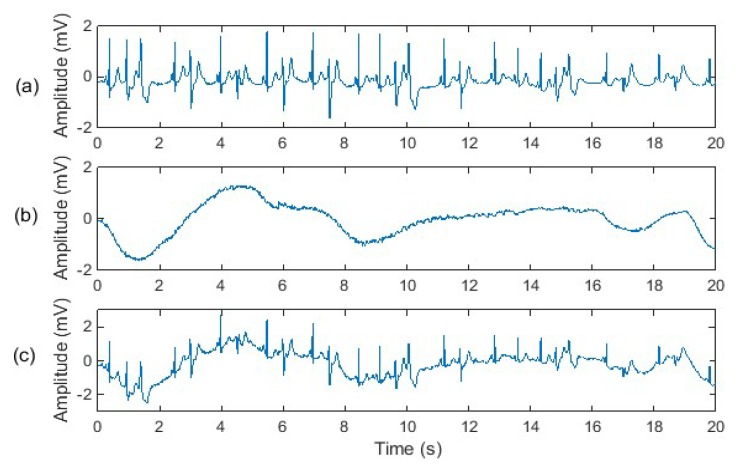
Experimental signals: (**a**) abnormal ECG from the MIT-BIH database “mitdb/106” where the premature ventricle contraction and ventricle couplet happens; (**b**) baseline wander noise from the MIT-BIH database “nstdb/bw”; (**c**) noisy ECG signal.

Two “clean” ECG signals were used in our experiment; one “clean” ECG signal which was a normal ECG recorded from a healthy adult, and another “clean” ECG signal was an abnormal ECG recorded from an adult with heart disease. An important reason why we used abnormal ECG signals in our experiment is that some heart diseases are reflected in the low-frequency portion of the ECG signal, which happens to overlap with the spectrum of motion artefacts. Hence, improper denoising methods may distort ECG waveforms while filtering out motion artefacts, leading to misdiagnosis of heart disease. Since a normal ECG signal does not contain the low-frequency features caused by heart disease, it is difficult to evaluate the effects of different denoising methods on low-frequency abnormal ECG features. Therefore, we use both a normal ECG and an abnormal ECG in our experiments. Synthetic motion artefact-corrupted ECG signals are shown in Figure 6 and Figure 7.

### 3.2. Evaluation Parameters

For the evaluation of noise reduction performance of different signal-processing techniques, various methods are being used in the literature. These methods can be divided into two main groups: subjective methods and objective methods. Subjective methods are based on the assessment of ECG signal quality by cardiologists or other experts while objective methods are based on mathematical equations, and hence there is no need for expert human assessment. The subjective methods for ECG quality evaluation are medically accepted [43,44], so in our experiment the denoised ECG signals were presented for subjective observation. Objective methods were also used to evaluate quantitatively the performance of different denoising methods. The following evaluation parameters were used in this work.

#### 3.2.1. Correlation Coefficient

The Pearson correlation coefficient is used here to measure the linear correlation of the filtered ECG signal *Y* and the “clean” ECG signal *X*:

(7) $$\rho_{X,Y}=\frac{E\left(\left(X-\mu_{X}\right)\left(Y-\mu_{Y}\right)\right)}{\sigma_{X}\sigma_{Y}}$$

where $\mu_{X}$ and $\sigma_{X}$ are the mean and standard deviation of the “clean” ECG signal; $\mu_{Y}$ and $\sigma_{Y}$ are the mean and standard deviation of the filtered ECG signal; *E* is the expectation. The result $\rho_{X,Y}$ falls between +1 and −1, where 1 represents an absolute positive linear correlation, 0 represents no linear correlation, and −1 represents an absolute negative linear correlation.

#### 3.2.2. Mean Squared Error (MSE)

Mean squared error (MSE) measures the average squared difference between the filtered ECG signal *Y* and the “clean” ECG signal *X*:

(8) $$MSE=\frac{1}{N}\overset{N}{\underset{i=1}{\sum }}{\left(X_{i}-Y_{i}\right)}^{2}$$

where *X_i_* is the “clean” ECG signal, *Y_i_* is the filtered ECG signal. The result MSE is always non-negative, and values closer to zero are better.

#### 3.2.3. R-Square (*R*^2^)

R-square (*R*^2^) is usually used to evaluate the goodness of fit between the two signals. In our study, we use this parameter to evaluate the fidelity of the filtered ECG signal:

(9) $$R^{2}=1-\frac{\sum_{i=1}^{N}{\left(X_{i}-Y_{i}\right)}^{2}}{\sum_{i=1}^{N}{\left(X_{i}-E\left(X\right)\right)}^{2}}$$

where *X_i_* is the “clean” ECG signal, *Y_i_* is the filtered ECG signal. R-square can take on any value between 0 and 1, and values closer to one are better.

#### 3.2.4. Signal-to-Noise Ratio (SNR)

The improvement of the signal-to-noise ratio (SNR) is defined as:

(10) $$Improvement\;of\;SNR=10\mathrm{lg}\left(\frac{\sum^{\;}s{\left(n\right)}^{2}}{\sum^{\;}{\left(\hat{s}\prime \left(n\right)-s\left(n\right)\right)}^{2}}\right)-10\mathrm{lg}\left(\frac{\sum^{\;}s{\left(n\right)}^{2}}{\sum^{\;}{\left(\hat{s}\left(n\right)-s\left(n\right)\right)}^{2}}\right)$$

where *s*(*n*) is the “clean” ECG signal, $\hat{s}\left(n\right)$ is the noisy ECG signal, and ${\hat{s}}^{\prime }\left(n\right)$ is the filtered ECG signal. A higher SNR value indicates that the filter has better performance in terms of noise reduction.

## 4. Experimental Results 

**Figure 8:**
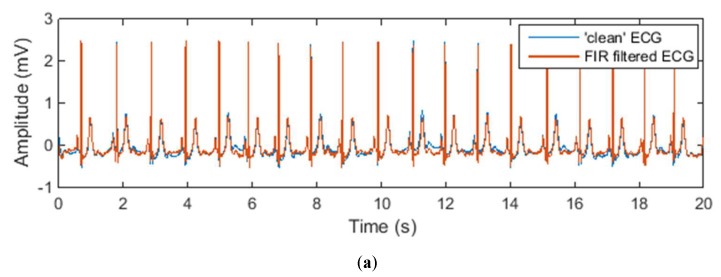

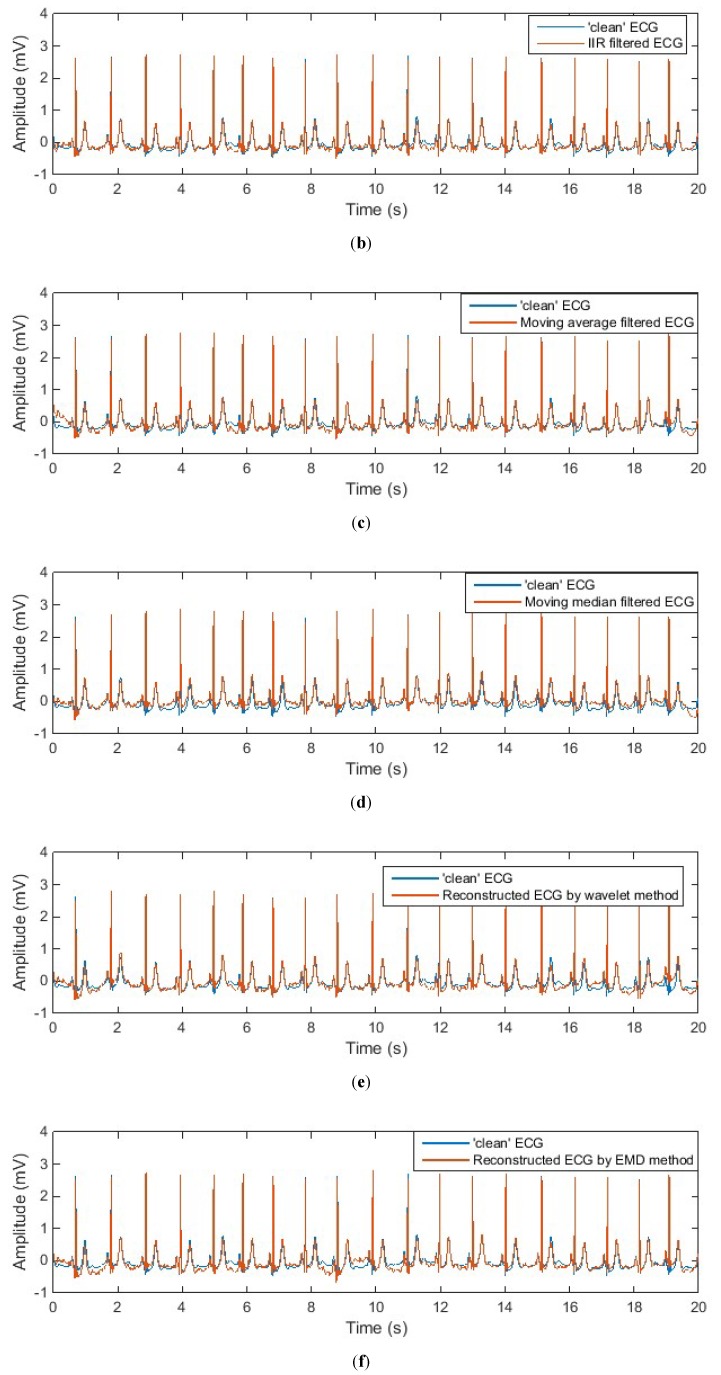

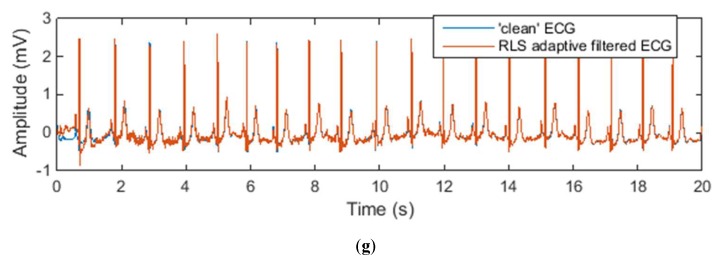
Denoised normal ECG waveform by different noise reduction methods: (**a**) FIR filter; (**b**) IIR filter; (**c**) moving average filter; (**d**) moving median filter; (**e**) wavelet transform denoising; (**f**) empirical mode decomposition (EMD); (**g**) adaptive filter.

**Figure 9:**
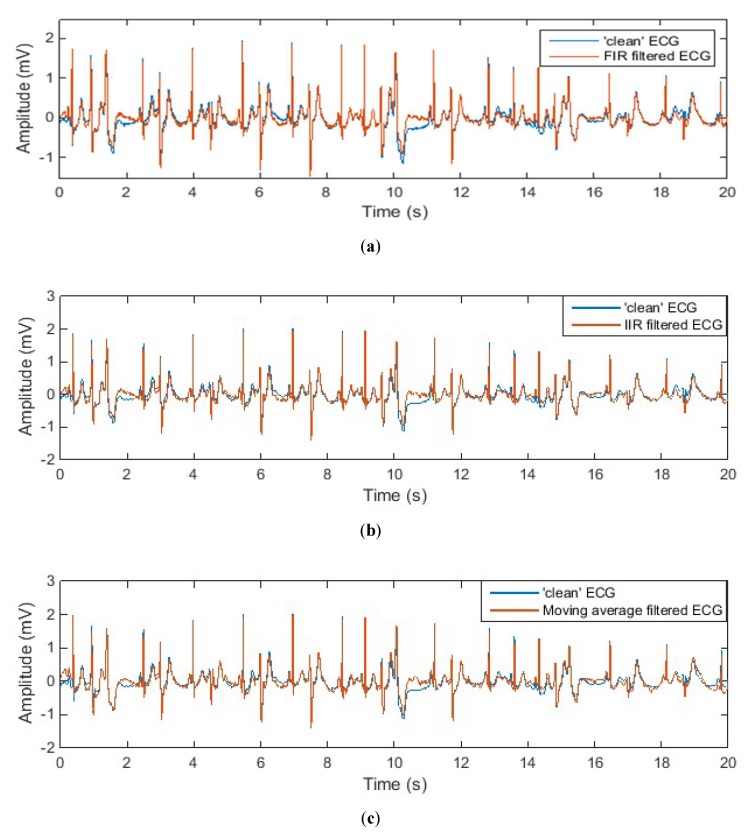

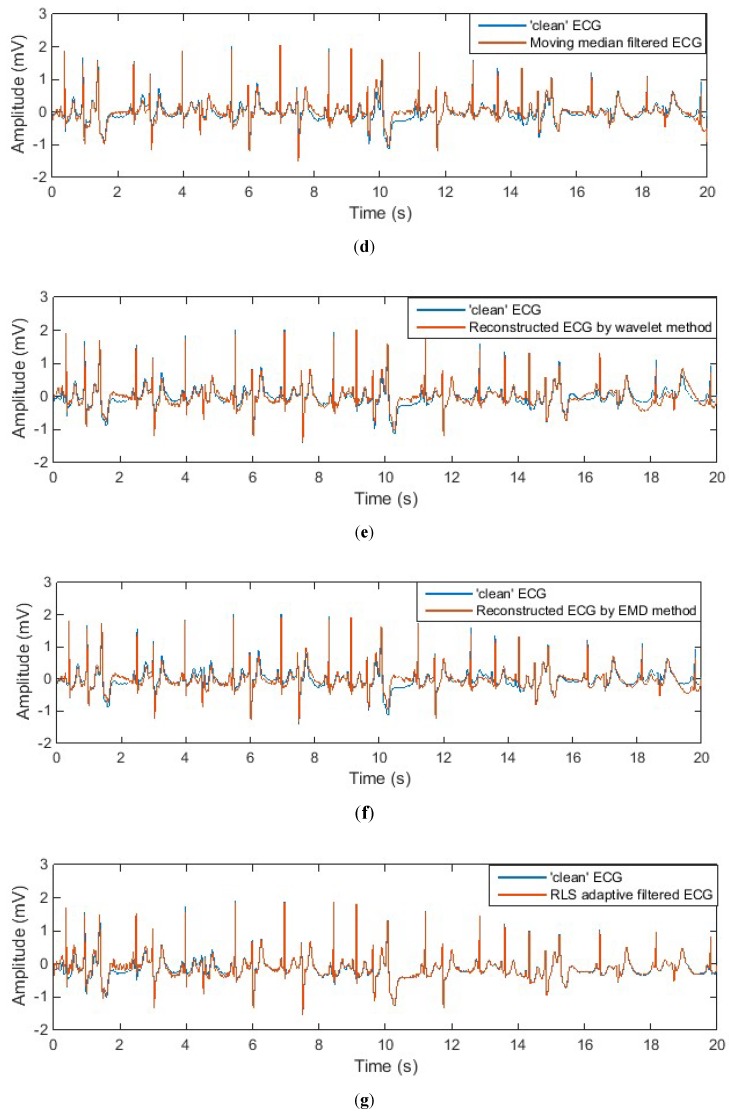
Denoised abnormal ECG waveform by different noise reduction methods: (**a**) FIR filter; (**b**) IIR filter; (**c**) moving average filter; (**d**) moving median filter; (**e**) wavelet transform denoising; (**f**) EMD; (**g**) adaptive filter.

Figure 8 and Figure 9 show the denoised ECG signal processed by different noise-reduction methods in comparison with the “clean” signal. It can be observed that different noise-reduction methods have similar performance when the input signal is a normal ECG waveform. But when it comes to an abnormal ECG waveform, all other methods except the adaptive filtering method cause the filtered ECG waveform to have certain degree of distortion. Take the ECG signal processed by the IIR filter as an example. The denoised ECG signal in Figure 8b perfectly overlaps the “clean” ECG signal, but the denoised ECG signal in Figure 9b does not completely overlap the “clean” signal. This shows that for normal ECG signal without low-frequency components, the IIR filter can successfully eliminate the noise without distorting the ECG waveform. However, for abnormal ECG signals containing low-frequency components, the IIR filter will filter out the low-frequency components of the abnormal ECG waveform while filtering the noise, resulting in distortion of the ECG waveform. From Figure 8g and Figure 9g it can be seen that whether the ECG signal is normal or abnormal, the filtered ECG signal completely overlaps the “clean” ECG signal, which indicates that the adaptive filter has outstanding performance in motion artefact reduction.

The observed results in the figures are consistent with the results of the evaluation parameters shown in Table 1 and Table 2. For both normal and abnormal ECG waveforms, the RLS adaptive filtering method outperforms other methods, with the highest correlation coefficient ($\rho_{X,Y})$ and *R*^2^, the smallest mean squared error (MSE), and the greatest improvement of signal-to-noise ratio (SNR). Especially in the case of the abnormal ECG waveform, the adaptive filter has significantly improved SNR over other noise-reduction methods. Specifically, IIR filtering improves the SNR by 15 dB, while RLS adaptive filtering improves the SNR by 27 dB. According to the subjective and quantitative evaluation results, the adaptive filter has excellent performance in reducing motion artefacts because it can perfectly preserve the morphology of the ECG waveform whilst successfully suppressing baseline wander.

## 5. Implementation of Adaptive Motion Artefact Reduction in Practice

The results from the above experiment showed that the adaptive filtering method is superior to other denoising methods in terms of motion artefact reduction. But it should be noted that the above experimental results are based on the use of synthesized noisy ECG signals. In actual ECG measurements, it is not possible to obtain a reference input signal for the adaptive filter that is identical to the noise in the measured ECG. However, in practical applications, we can choose a signal with a high correlation with motion artefacts as the reference input signal of the adaptive filter, because the performance of the adaptive filter on the reduction of motion artefacts mainly depends on the correlation between the reference input signal and the noise.

In fact, the need for a reference input signal limits the application of adaptive filters for noise cancellation, as this usually means that specially designed hardware and related firmware are required to support the provision of the reference input signal. Although the need for a reference input signal poses a challenge to using adaptive noise cancellation, some researchers have managed to investigate the possibility of using auxiliary sensors for providing a reference input signal to adaptive filters for reducing the motion artefact in ECG signals [36,45,46,47,48,49]. In these studies various sensors such as strain sensors, accelerometers, and optical sensors have been used as the source of reference input signal in adaptive filters.

**Figure 10:**
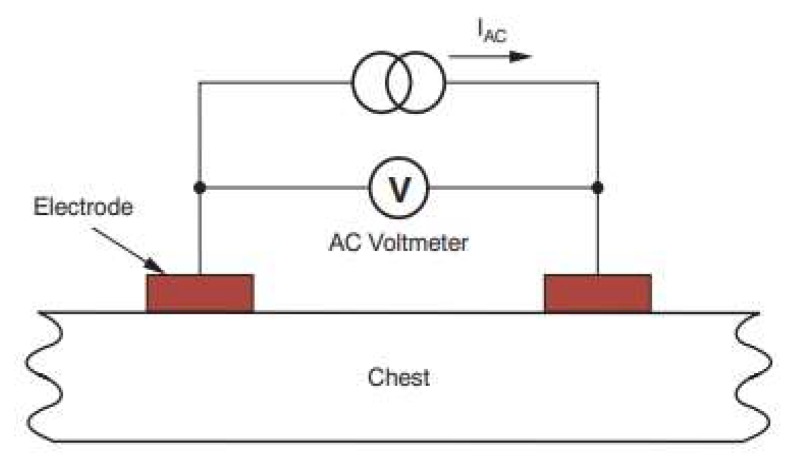
The principal of impedance pneumography (IP) measurement.

In this paper, we use the impedance pneumography (IP) signal as a reference input signal for adaptive filtering, because the IP signal can be measured by the same pair of electrodes that measure ECG signals so that the motion that affects the ECG signal will also affect the IP signal. IP is a commonly used technique to monitor respiration. It measures changes in the electrical impedance of the person’s thorax caused by respiration. An increase in the volume of air respiration during each breathing cycle reduces the electrical conductivity of the human thorax. The implementation of IP can be done by a two-electrode method, as demonstrated in Figure 10. In this method, a low-amplitude high-frequency current is injected into the tissue through the electrodes, and then the voltage gradient between the electrodes is measured.

**Figure 11:**
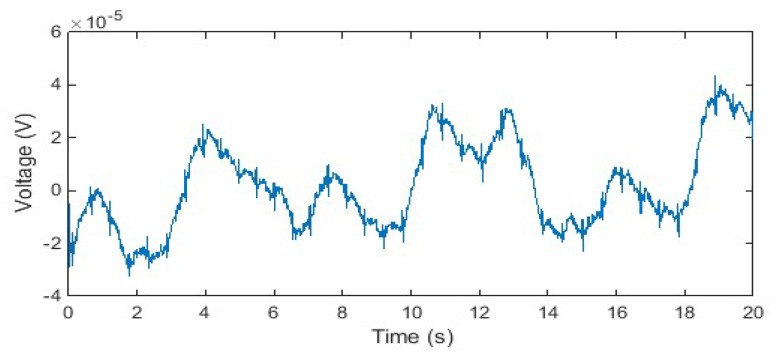
The IP signal consisting of respiratory signal and motion artefacts.

The IP signal is a measure of the electrical impedance change of the subject’s thorax caused by respiration. From the perspective of the ECG signal, the IP signal reflects information about the respiratory motion artefact due to chest movement during breathing. In addition, the measured IP signal also contains information about other motion artefacts that affect the ECG signal because both the IP and ECG signals are measured using the same pair of electrodes at the same electrode location, as demonstrated in Figure 11.

**Figure 12:**
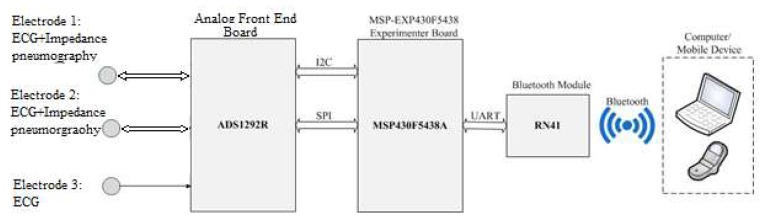
Hardware configuration.

**Figure 13:**
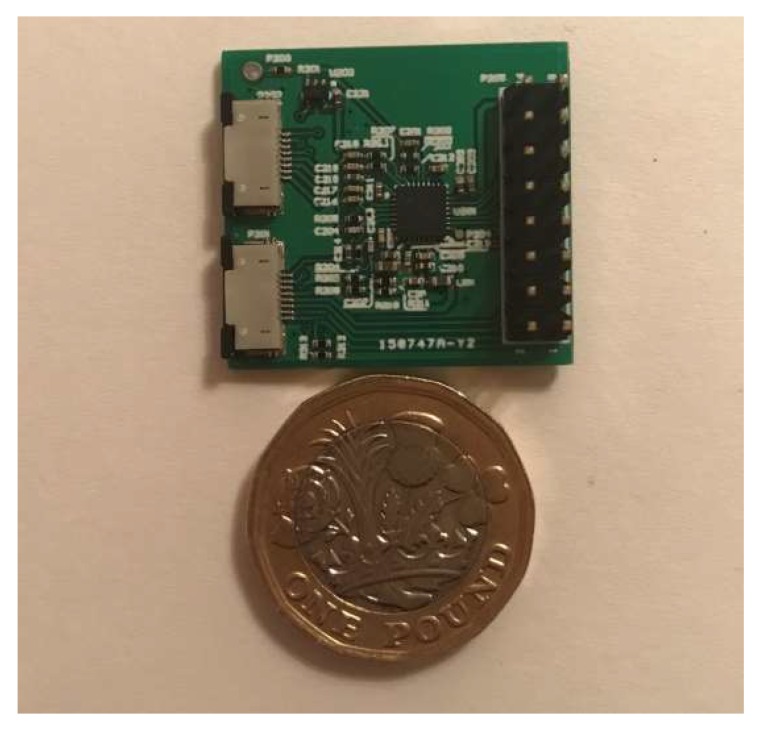
The analog front-end (ECG + IP functions).

Because of these characteristics of the IP signal, we propose that it is a good source of reference input signal for adaptively reducing motion artefacts of the ECG signal during body moving/exercising. In order to verify the performance of adaptive filtering in motion artefact reduction in practice, and to investigate the possibility of using the IP signal as the reference input signal for adaptive filtering, we designed special hardware to measure the ECG signal and the required reference signal. The hardware configuration is shown in Figure 12. The hardware supports the simultaneous measurement of ECG signals and IP signals using the same pair of electrodes. It consists of a microcontroller MSP430F5438A (Texas Instruments, Dallas, TX, USA), an analog front-end ADS1292R (Texas Instruments, Dallas, TX, USA), a Bluetooth module RN41 (Microchip, Chandler, AZ, USA), and three electrodes. The ADS1292R chip is a low-power, 2-channel analog front-end. Its channel 1 supports IP measurement and channel 2 supports ECG measurement, as demonstrate in Figure 13. In the IP measurement, a modulation block in the chip produces a 2.42 V, 32 kHz square wave and injects it into the body as a carrier signal that is amplitude-modulated by a low-frequency signal generated as a result of the breathing action. In our application, a pair of electrodes is used, but wired into both channel 1 and channel 2 for simultaneous measurement of ECG and IP signals.

**Figure 14:**
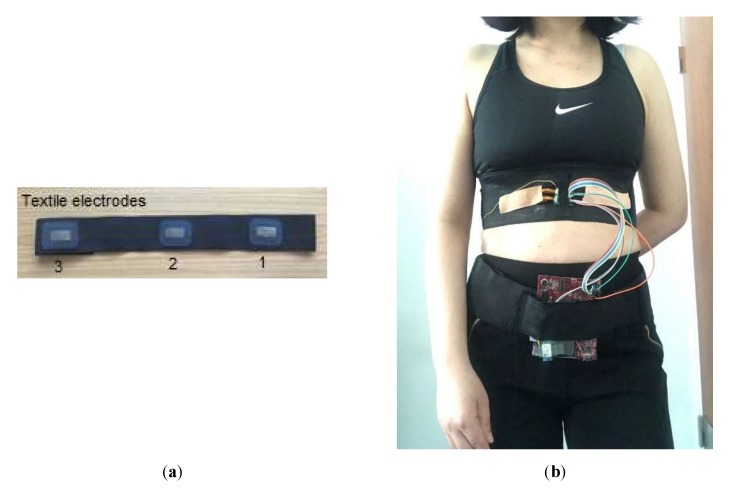
(**a**) The arranged textile electrodes, (**b**) the measurement of the ECG and IP signals.

Figure 14 demonstrates the electrodes that are used in the ECG and IP signals’ measurement. The electrodes are textile based and made of conductive fabric (MedTex P-130, Shieldex, Bremen, Germany). The fabrication of the textile electrodes and their performance for ECG measurement has been discussed in another of our articles [50]. The electrodes are integrated in an elastic band so that they can easily and comfortably be placed on the wearer’s chest by the pressure of the belt. During measurement, the electrode belt was placed on the horizontal line of the 6th intercostal space, in which electrodes 1 and 2 measured the ECG and IP signals and were placed on the left and right mid-clavicular line, respectively, whilst electrode 3 is used to reduce common mode noise and was placed on the patient’s back. In order to obtain motion artefact-contaminated ECG signals, in this exploratory experiment we trigger a motion artefact by pressing one of the electrodes while recording the ECG and IP signals.

**Figure 15:**
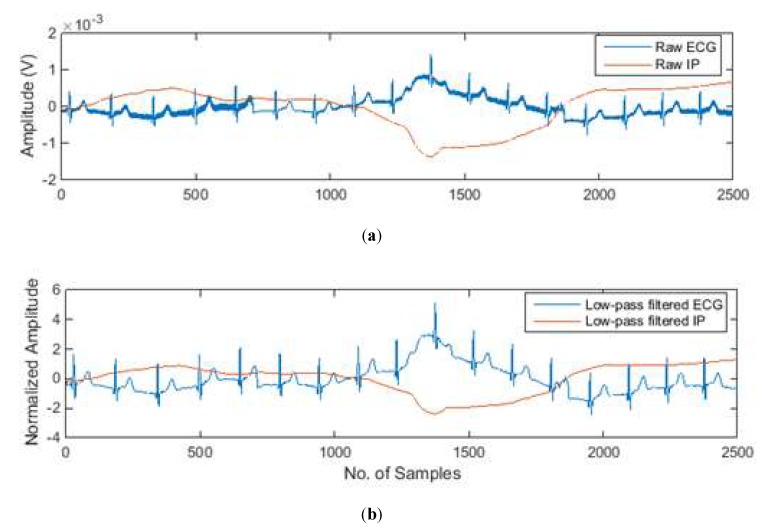
(**a**) The motion artefact-contaminated ECG and IP signals; (**b**) the low-pass filtered and normalized ECG and IP signals.

Figure 15a shows the ECG and IP signals measured by our hardware design. The presented signal was raw, unfiltered and was measured on a middle-aged healthy female. It can be noticed that the measured ECG is contaminated by power line interference and the motion artefact. In order to eliminate the power line interference and its harmonic, a 40 Hz low-pass IIR Butterworth filter and zero-phase filtering technique are used to process the raw signals. After low-pass filtering, ECG and IP signals were then normalized by Z-score normalization to unify their amplitude units, as shown in Figure 15b.

The absolute value of the correlation coefficient between the ECG signal and the IP signal in Figure 15 is 0.8, which indicates that there is a strong correlation between the motion artefact-contaminated ECG signal and the IP signal. In adaptive filtering, the ECG and IP signals are both fed into an RLS adaptive filter, the defaulted filter length is 32, and the forgetting factor is 1.

**Figure 16:**
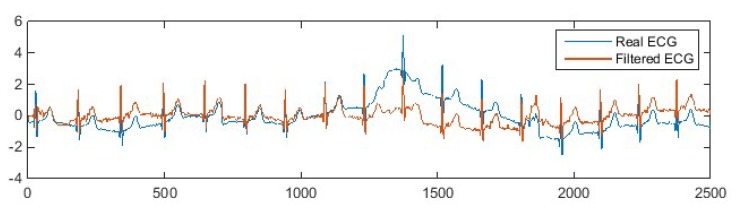
The denoised ECG signal by the adaptive filter.

The output of the adaptive filtered ECG signal is shown in Figure 16.

As can be observed from Figure 16, the adaptive filter successfully reduced most of the motion artefacts and kept the ECG waveform undistorted. The result proves that the adaptive filtering technology can effectively reduce motion artefacts in practical wearable ECG measurement.

## 6. Discussion

We studied the performance of different signal-processing techniques in reducing motion artefacts in ECG signals. Our investigations prove that the zero-phase IIR filter and the adaptive filter have a better performance in motion artefact reduction than other noise-reduction methods. We have found that adaptive filters in particular exhibit excellent performance in reducing motion artefacts in abnormal ECG waveforms.

The performance of adaptive filtering in actual ECG measurement is also evaluated with ECG and IP signals measured by our hardware design. In our experiment, the impedance pneumography signal that is measured by the same pair of ECG electrodes has a good correlation with the ECG disturbed by motion artefacts, so the adaptive filter performs well in reducing such motion artefacts. This is mainly because the impedance pneumography signal contains not only the information about the chest movement caused by breathing, but also artefacts caused by body movement, which happen to coincide with the composition of motion artefacts. The results from our study demonstrate that the adaptive filter can be used in motion artefact reduction in wearable end uses, provided that the reference input signal has a high correlation with the noise to be suppressed.

In fact, in actual ECG measurement, the adaptive filter should have better motion artefact reduction performance than the high-pass IIR filter, especially for abnormal ECG waveforms measured from patients with heart disease. This is because the high-pass IIR filter specifies a cut-off frequency (0.5 Hz is the widely accepted cut-off frequency in practice), which means only the frequency components of the motion artefact bellow the specified cut-off frequency are eliminated. However, the main frequency range of most of the motion artefacts are wider than 0.5 Hz, so a 0.5 Hz high-pass filter is not sufficient to eliminate most of the motion artefacts. Moreover, when the abnormal ECG waveform has a frequency component lower than 0.5 Hz, a 0.5 Hz high-pass filter will cause the loss of abnormal information represented by this frequency component. Apparently, the adaptive filter does not have the concerns that the high-pass IIR filter has. The adaptive filter does not restrict the cut-off frequency of the filter because it adaptively adjusts its coefficient based on the input reference signal and the adaptive algorithm used. In fact, the adaptive filter obtains an estimated noise signal through an adaptive algorithm, and then subtracts the estimated noise from the noisy ECG signal to obtain a denoised signal. This is why the adaptive filter can eliminate motion artefacts whose frequency components overlap with the ECG waveform without distorting the ECG waveform.

The concern with using adaptive filtering techniques to reduce motion artefacts from ECG signals is that the reference input signal used should be highly correlated with the motion artefact. Therefore, in practical applications, the reference input signal should be selected carefully to ensure the performance of adaptive filter in reducing motion artefact for wearable end uses. Our experiments showed that the impedance pneumography signal has good correlation with motion artefacts generated by pressing the electrode. In reality, motion artefacts are generated by different motions, so the correlation between IP signals and motion artefacts needs further study. In general, before feeding the IP signal to the reference input of the adaptive filter, the correlation between the IP signal and the noisy ECG signal should be evaluated. Only when the IP signal has good correlation can it be used for adaptive filtering, otherwise the performance of the adaptive filter will be compromised. Although we have found high correlations (≥0.8) between IP signals and motion artefacts, we will be conducting extensive further experiments in order to deploy our method to wearable devices for online ECG monitoring.

## Figures and Tables

**Table 1:** The performance of different noise reduction methods (normal ECG waveform).

Normal ECG Waveform				
	Correlation $\rho_{X,Y}$	MSE	*R* ^2^	Improvement of SNR (dB)
FIR filter	0.9796	0.0072	0.9571	17.6449
Zero-phase IIR filter	0.9806	0.0064	0.9611	18.1149
Moving average filter	0.9681	0.0108	0.9343	15.8381
Moving median filter	0.9732	0.0161	0.9025	14.1233
Wavelet	0.9643	0.0125	0.9244	15.2303
EMD	0.9656	0.0122	0.9262	15.3350
RLS Adaptive	0.9877	0.0042	0.9750	19.9919

**Table 2:** The performance of different noise reduction methods (abnormal ECG waveform).

Abnormal ECG Waveform				
	Correlation $\rho_{X,Y}$	MSE	*R* ^2^	Improvement of SNR (dB)
FIR filter	0.9450	0.0130	0.8925	15.0486
Zero-phase IIR filter	0.9456	0.0128	0.8939	15.1285
Moving average filter	0.9435	0.0135	0.8879	14.8869
Moving median filter	0.9349	0.0158	0.8684	14.1955
Wavelet	0.9335	0.0162	0.8658	14.1082
EMD	0.9285	0.0172	0.8574	13.8443
RLS Adaptive filter	0.9750	0.0061	0.9491	27.4151
